# Sensors Layout Optimization Design of Rocket Sled Test System

**DOI:** 10.3390/s24113641

**Published:** 2024-06-04

**Authors:** Hongjun Qian, Wenjie Wang, Xu Zhao, Yi Jiang

**Affiliations:** School of Aerospace Engineering, Beijing Institute of Technology, Beijing 100081, China; 3220215020@bit.edu.cn (H.Q.);

**Keywords:** rocket sled test system, sensor layout, shock wave interference, acoustic directivity

## Abstract

The rocket sled, as a ground dynamic test system, combines the characteristics of the wind tunnel test and the flight test. However, some practical factors, such as shock wave interference, ground effect, and high-intensity aerodynamic noise will cause serious interference and even failure of the uniformly distributed sensors during horizontal sliding in a wide speed range. The AGARD HB-2 standard model is employed as the payload to simulate the aerodynamic and aeroacoustic characteristics during the variable acceleration period, aiming to optimize the test sensors layout. It is observed that in the high Mach number flow fields, strong coupling behaviors among complex waves will occur. The peak of wake vortex strength will appear at 1.5 s and gradually diminish over time. In addition, when the vortex between the load and the booster is monitored, its position shifts forward in the subsonic stage, then gradually moves backward and expands in the supersonic stage. Acoustic directivity is pronounced at subsonic and transonic speeds, pointing towards 75° and 135° relative to the sliding speed, respectively. These results can provide technical support for sensor layout and high-precision testing in rocket sled tests.

## 1. Introduction

**Figure 1:**
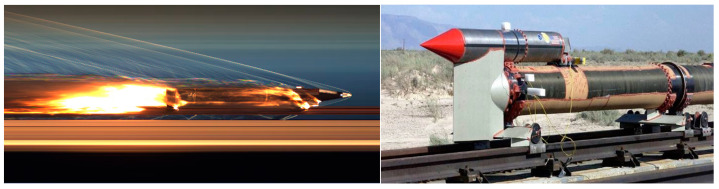
Rocket sled test system.

The rocket sled test system has overcome many defects of wind tunnel and flight tests and is widely used in current aircraft testing [1,2]. A rocket sled system usually consists of a rocket engine, payload, slippers, and rails, as shown in the Figure 1. This test setup employs solid or liquid rocket engines to power the sled, carrying the test object along a high-precision track for data acquisition [3,4]. The rocket sled test provides valuable data on the performance of the payload under different velocities and accelerations. A single test generates a substantial volume of experimental data, providing comprehensive information for analysis and evaluation. The rocket sled has become an indispensable tool in fields such as escape tower, parachute testing [5], verifying new materials and structures, etc. Additionally, as one of the single-stage-to-orbit launch methods, the rocket sled can fully use its propulsion characteristics to provide high takeoff velocity for spacecraft. This pattern increases the transporting capacity of space vehicles and enables a reusable launch method [6].

The high sliding speed of the rocket sled introduces disturbances such as wave interference, jet effect, and aerodynamic noise, which influence the flow field around the payload. These factors may induce structural resonance and fatigue damage, potentially shortening the lifespan of the payload. Moreover, some sensors should be placed in areas less susceptible to disturbances. Prior simulation analysis of the aerodynamic and aeroacoustic characteristics of the rocket sled system can provide insights for optimizing the sensors’ layout.

Plenty of research has been conducted on the complex flow field characteristics of the rocket sled system. Rigali et al. [7] carried out experiments on a monorail rocket sled system, and the results suggested that the sled-testing technique appears to be the most promising method for simulating low-altitude flight conditions. The study, guided by experimental design principles, developed models that quantify the predicted behavior of a test article when the actual properties differ from the modeled properties [8]. Establishing the boundary conditions for simulating the rocket sled, Zhang et al. [9,10] demonstrated the effectiveness of combining finite element analysis with multisystem theory. Soft sled tests conducted at the Holloman High Speed Test Track (HHSTT) showed that the vibration levels measured in soft sled tests were a factor of 2× softer than Maglev in the lateral direction, and a factor of 3× softer in the vertical direction [11]. Terrazas et al. [12] developed a predictive model for the HHSTT sled tests at various velocity regimes and simulated the water braking process. Wang et al. [13] found that in the flow field of the anti-D-shaped rocket sled, the unsteady large-scale vortex and shock motions appeared due to the strong momentum transfer, and the axisymmetric geometry was suitable for the rocket sled. A rocket sled test with a rotating detonation engine (RDE) was conducted by Goto et al. [14]. The combustor pressure and the specific impulse of the sled test were comparable to the results of the laboratory stand firing test using a load cell. The aerodynamic and aeroacoustic characteristics of a dual-rail subsonic rocket sled were simulated by Yan et al. [15]. They found that the vortices between the two connectors were one of the main sources of aerodynamic noise. Zhou et al. [16] determined the relationship among temperature, strain rate, and strain at the onset of rail gouging, through experimental and theoretical analyses. A dynamic grid method was used to simulate an axisymmetric slender rocket sled (ASRS) and a reversed waverider rocket sled (RWRRS) by Yu et al. [17]. They found that the RWRRS was able to increase lift and reduce drag, and the ability of the RWRRS to reduce pressure oscillation was confirmed by comparing the sound pressure level value.

The current research is mainly limited to either the subsonic or the supersonic phase. However, the rocket sled test system is a dynamic process that accelerates from standstill to high speed. Additionally, there is minimal research on the sensors’ layout in rocket sled test systems. The present work simulates the variable acceleration process of the rocket sled. The changes in velocity, pressure, and vortex position and size over time are analyzed. In addition, different sliding velocities are studied to obtain the alterations in sound pressure level distribution and acoustic directivity. The results can serve as a reference for the sensor layout in the rocket sled test system.

## 2. Model Setup

**Figure 2:**
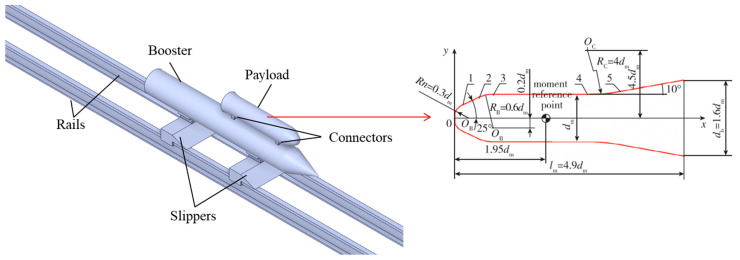
Schematic of rocket sled system and dimensions of AGARD HB-2.

The geometric model used in the simulation is shown in Figure 2. The payload is connected to the booster, ensuring stability and safety during high-speed sliding. The rocket sled is placed on the rails through the slippers, enabling the smooth sliding of the test system. Additionally, the numerical simulation utilizes the optimized AGARD HB-2 standard model as the payload. The AGARD HB-2 standard model is a classical sphere–cylinder–skirt configuration [18,19], as shown on the right side of Figure 2. Its flow field can cover subsonic, transonic, and supersonic speed regimes at specific blowing velocities. At the juncture between the cylinder and the skirt, boundary layer separation occurs. Bow shock waves and separated shock waves coexist at the head, accompanied by corner expansion phenomena [20].

## 3. Computational Methods and Validations

### 3.1. Numerical Method

The compressible Navier–Stokes equations adopted in the present work are given as follows:

(1) $$\frac{\partial \rho }{\partial t}+\nabla \cdot \left(\rho U\right)=0$$

(2) $$\frac{\partial \left(\rho U\right)}{\partial t}+\nabla \cdot \left(\rho UU\right)=\nabla \cdot \left(-pI+\hat{T}\right)$$

(3) $$\frac{\partial \left(\rho E\right)}{\partial t}+\nabla \cdot \left(\rho EU\right)=\nabla \cdot \left(-\dot{q}+\left(-pI+\hat{T}\right)\cdot U\right)$$

where ρ, U, and *p* are the density, velocity vector, and pressure of the fluid, respectively. $E=e+\frac{{\left|U\right|}^{2}}{2}$ is the total energy of the fluid, *e* is the specific internal energy of the fluid. $\hat{T}$ is the viscous stress tensor, and $\dot{q}$ is the heat flux vector.

The Shear Stress Transport (SST) k-ω model [21] is used to simulate the turbulence, where k is the turbulent kinetic energy and ω the specific turbulent dissipation rate. The SST k-ω model is as follows:

(4) $$\frac{\partial }{\partial t}(\rho k)+\frac{\partial }{\partial x_{j}}\left(\rho ku_{i}\right)=\frac{\partial }{\partial x_{j}}\left(\Gamma_{k}\frac{\partial k}{\partial x_{j}}\right)+G_{k}-Y_{k}$$

(5) $$\frac{\partial }{\partial t}(\rho \omega )+\frac{\partial }{\partial x_{j}}\left(\rho \omega u_{i}\right)=\frac{\partial }{\partial x_{j}}\left(\Gamma_{\omega }\frac{\partial \omega }{\partial x_{j}}\right)+G_{\omega }-Y_{\omega }+D_{\omega }$$

It utilizes the original k-ω model in the sub- and log-layer and gradually switches to the standard k-ε model in the wake region of the boundary layer. The k-ε model is also used in free shear layers. Additionally, a modification to the eddy viscosity has been introduced. The SST model leads to a significant improvement for all flows involving adverse pressure gradients. The SST k-ω model is reliable for a wide class of flows (for example, adverse pressure gradient flows, airfoils, transonic shock waves).

An implicit pressure-based solver suitable for supersonic flow is employed [22]. The pressure-velocity coupling scheme utilizes a coupled approach. All the convection terms in transport equations are discretized by a second-order upwind scheme, providing adequate fidelity for engineering applications, and the gradient is evaluated by the least squares of the corresponding variable at the neighbor cell centers.

The Ffowcs Williams and Hawkings (FW-H) equation is used for the acoustic solution, which can be derived by manipulating the continuity equation and the Navier–Stokes equations. The FW–H equation is analytically superior for aeroacoustics because it is based on the conservation laws of fluid mechanics rather than on the wave equation [23]. Thus, the FW–H equation is valid even if the integration surface is in the nonlinear region. In addition, the approach is less sensitive to the placement of the integration surface if the quadrupole is neglected. The FW-H equation can be written as follows [23,24]:

(6) $$\frac{1}{a_{0}^{2}}\frac{\partial^{2}\rho^{\prime }}{\partial t^{2}}+\nabla^{2}p^{\prime }=\frac{\partial^{2}}{\partial x_{i}\partial x_{j}}\left\{T_{ij}H(f)\right\}-\frac{\partial }{\partial x_{i}}\left\{\left[P_{ij}n_{j}+\rho u_{i}\left(u_{n}-v_{n}\right)\right]\delta (f)\right\}+\frac{\partial }{\partial t}\left\{\left[\rho_{0}v_{n}+\rho \left(u_{n}-v_{n}\right)\right]\delta (f)\right\}$$

where $u_{i}$ is the fluid velocity component in the direction, $u_{n}$ is the fluid velocity component normal to the surface *f* = 0, $v_{n}$ is the surface velocity component normal to the surface. H(f) and δ(f) are the Heaviside function and the Dirac delta function, respectively, $p^{\prime }$ is the sound pressure at the far field ($p^{\prime }=p-p_{0}$), and $T_{ij}$ is the Lighthill stress tensor, defined as follows:

(7) $$T_{ij}=\rho u_{i}u_{j}+P_{ij}-a_{0}^{2}\left(\rho -\rho_{0}\right)\delta_{ij}$$

where $P_{ij}$ is the compressive stress tensor. For a Stokesian fluid, this relationship is given by the following:

(8) $$P_{ij}=p\delta_{ij}-\mu \left[\frac{\partial u_{i}}{\partial x_{i}}+\frac{\partial u_{j}}{\partial x_{i}}-\frac{2\partial u_{k}}{3\partial x_{k}}\delta_{ij}\right]$$

### 3.2. Computational Grid and Boundary Conditions

**Figure 3:**
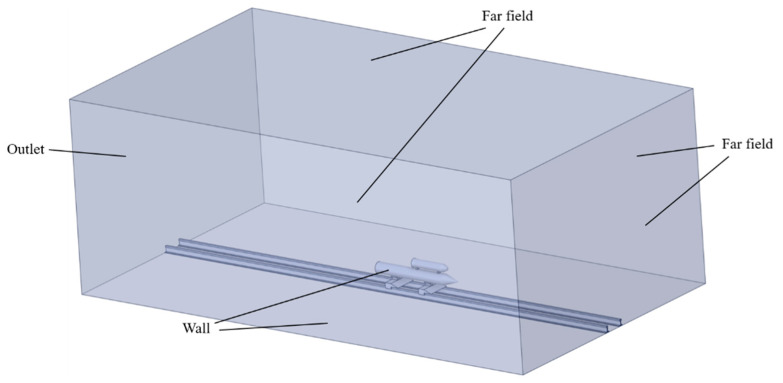
Computational field and boundary types.

**Figure 4:**
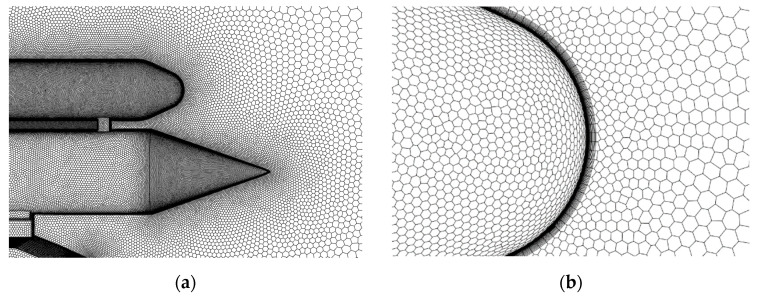
(**a**) Mesh generated for computation; (**b**) boundary layer of payload head.

The computational field and boundary conditions are illustrated in Figure 3. The width of the field is set to 20 times the diameter of the payload, and the length is set to 16 times the length of the payload. The pressure far-field conditions simulate the sliding motion of the rocket sled. The boundary conditions for the simulation are used with an atmospheric temperature of 300 K and an atmospheric pressure of 101,325 Pa. Polyhedral cells are used to fill the computational field, as shown in Figure 4. The thickness of the first cell of the boundary layer is 1 μm, and the growth rate is 1.05. Grid refinement is applied to the regions near the payload and booster to guarantee computational accuracy. Additionally, during the simulation, an adaptive mesh is employed in areas of high-pressure gradients to distinguish shock waves.

**Figure 5:**
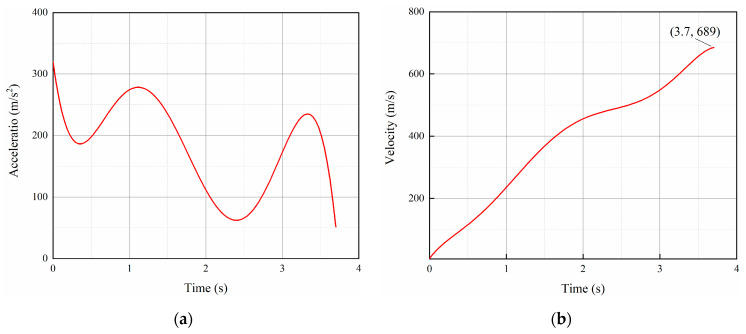
Rocket sled test result: (**a**) acceleration curve; (**b**) velocity curve.

The acceleration and velocity curves of the variable acceleration rocket sled sliding, fitted based on a specific rocket sled test result, are depicted in the Figure 5. The velocity function is input into the pressure far-field condition, defining the Mach number for the variable acceleration inflow. This setup enables the simulation of the rocket sled accelerating from standstill to Mach 2. It is observed that the acceleration is highest in the early stage of sliding, with an initial acceleration reaching 320 m/s^2^. At the end of the sliding, at 3.7 s, the velocity reaches its maximum value of 689 m/s.

### 3.3. Grid and Time Step Independence

**Figure 6:**
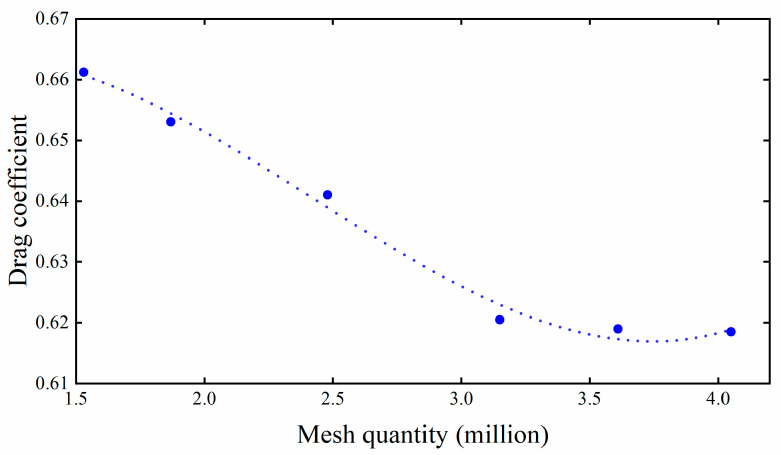
Grid independence.

Six different grid densities are employed to verify grid independence, whose cell numbers are 1.53 million, 1.87 million, 2.48 million, 3.15 million, 3.61 million, and 4.05 million, respectively. The drag coefficients of the rocket sled are shown in Figure 6. It can be observed that after reaching a quantity of 3.15 million, further grid refinement leads to fluctuations in the drag coefficient of less than 2%. Finally, a total number of 3.61 million cells are used in the numerical simulation, which is sufficient to ensure computational accuracy.

**Figure 7:**
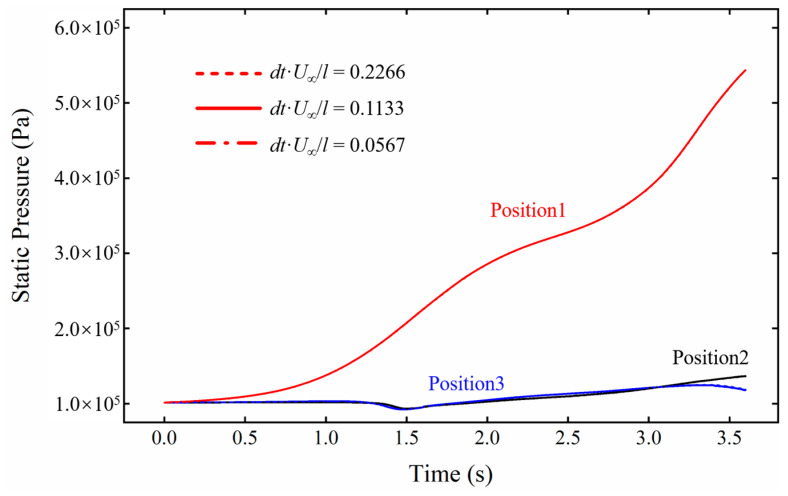
The variation of static pressure near the payload’s head under different computational time steps.

For transient simulations, in addition to ensuring grid independence, it is also necessary to determine the appropriate time step. A smaller time step within a suitable range can reduce the truncation error and improve computational accuracy. However, an excessively small time step may increase the round-off error and extend the simulation time. The non-dimensional time step is set as *dt·U_∞_*/*l* = 0.2266, 0.1133, 0.0567, respectively (where *U_∞_* is the freestream speed, and *l* is the length of the rocket sled). Figure 7 presents the static pressure at three positions around the payload with the three different time steps during sliding. All the curves for these time steps display approximately the same variation trend. Therefore, considering simulation cost and efficiency, the non-dimensional time step of *dt·U_∞_*/*l* = 0.1133 is selected for numerical simulations.

### 3.4. Validation of Numerical Methods

The T-38 wind tunnel, a 1.5 m × 1.5 m trisonic blow-down wind tunnel of the VTI Institute in Belgrade [25], is frequently utilized for testing the aerodynamic characteristics of various models. This test facility was designed and built by the Dilworth Secord Meagher and Associates Limited (DSMA) company [26]. For subsonic and supersonic tests, the test section has solid walls, while for transonic tests, a section with porous walls is used. The wall porosity can be adjusted between 1.5% and 8%, depending on the Mach number, to achieve optimal flow quality. In the test section, Mach numbers ranging from 0.2 to 4 can be achieved, with Reynolds numbers up to 1.1 × 10^8^/m. The test model is supported by a tail sting mounted on a pitch-and-roll mechanism, allowing for precise control of aerodynamic angles. The positioning accuracy is 0.05 degrees for both pitch and roll.

**Figure 8:**
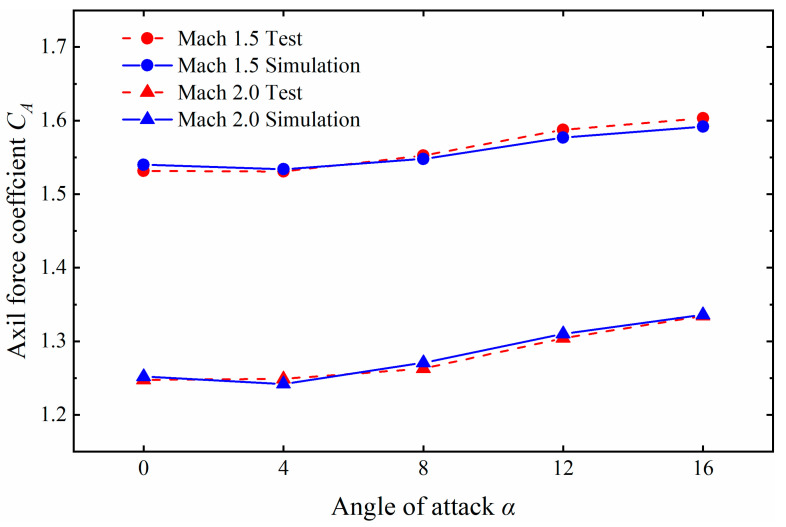
Comparison of simulation and test data.

To validate the numerical models adopted in this work, six cases for the AGARD HB-2 model, whose Mach numbers (1.5 and 2.0) and angles of attack (0°, 8°, 12°, and 16°) are the same as those in the wind tunnel tests in Ref. [27], have been investigated and examined. The results of the comparison of the simulations of the axil force coefficient (*C_A_*) and experimental measurement are illustrated in Figure 8. It can be seen that *C_A_*, obtained from the simulation, is in good agreement with the test results, with a deviation of less than 3%. The results manifest good agreement, which validates the numerical methods adopted.

## 4. Results and Discussions

### 4.1. Flow Field Analysis and Sensors Layout

**Figure 9:**
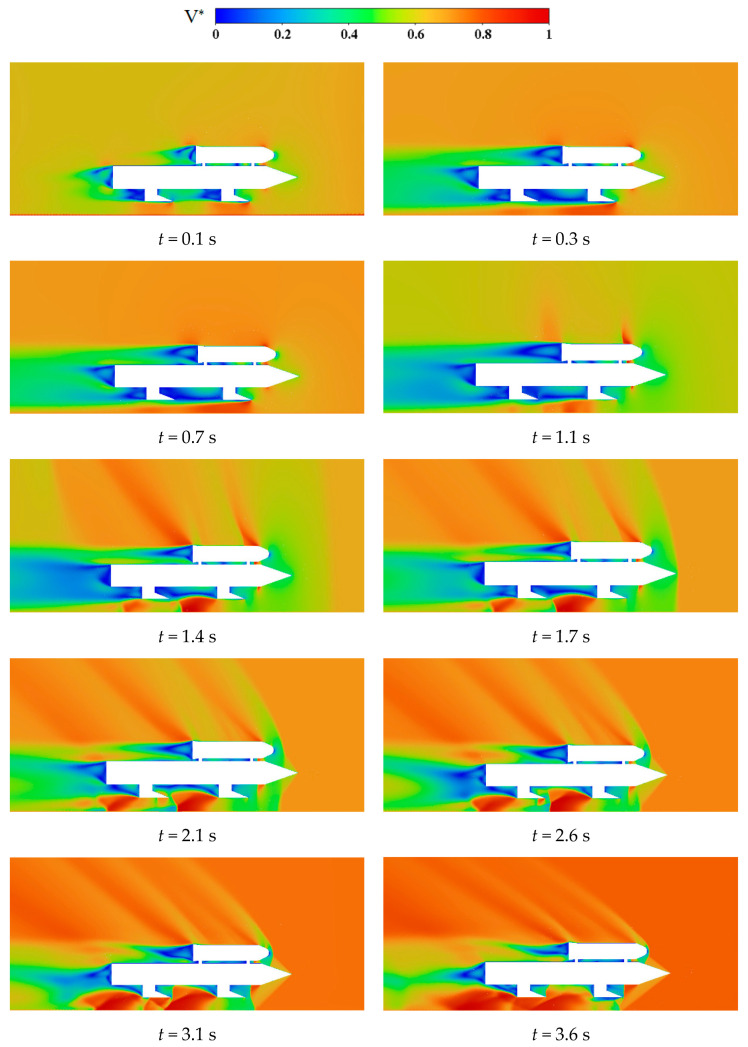
Velocity distributions on the symmetry plane.

The velocity distribution of the symmetry plane flow field of the variable acceleration rocket sled sliding from 0 s to 3.6 s is shown in Figure 9. The velocity is normalized dimensionless, $V^{*}=V_{L}/V_{\max}$ (where $V_{L}$ represents the velocity on the symmetry plane, and $V_{\max}$ represents the maximum velocity at this sliding moment). At the beginning of sliding, several low-speed regions form behind the rocket sled and between the two connectors. As the sliding time goes on, significant changes occur in the low-speed region at the rear of the payload. At 1.1 s, the region noticeably expands, but it rapidly shrinks after 1.4 s. It can be seen that after 1.4 s, multiple shock waves appear at the rear of the payload, causing interference in this low-speed area. Additionally, a high-speed airflow appears above the booster at 1.4 s. This airflow is likely caused by the shock wave generated by the front slipper, affecting the velocity distribution at the rear of the payload. This suggests that the influence of the payload wake vortex initially expands and then diminishes with increasing sliding time.

Then, the complex wave systems are analyzed from 1.1 s to 3.6 s. There is a notable high-speed region at the head of the payload and the booster, at 1.1 s, where air forms expansion waves, and there is a weak shock wave at the bottom of the front slipper. When the sliding time reaches 1.4 s, significant shock waves appear in front of the rocket sled, and the expansion waves at the head of the payload and the booster become more prominent. Moreover, there is a noticeable expansion wave below the front slipper, interfering with the shock waves generated by the rear slipper. As the sliding time progresses, the expansion wave below the rear slipper becomes significant, and the shock waves at the front of the payload and the booster begin to move backward. The shock waves at the front of the booster impinge on the payload’s head during the time from 2.1 s to 3.6 s. At 2.6 s, the shock waves from the booster’s head reflect off the ground and interfere with the expansion waves. The expansion wave generated by the rear slipper affects the vortex behind the booster. When the velocity reaches the maximum, there is extensive interference of waves between the rocket sled and the ground. In the area beneath the booster, a low-speed zone forms between the two slippers in the initial stage of sliding, reaching its maximum area at 0.7 s. As the speed increases, the expansion wave generated at the front slipper alters the velocity distribution in this region. By the end of the sliding phase, the low-speed zone is confined to the vicinity of the slippers, with the high-speed airflow dominating the entire area beneath the rocket sled. Based on the simulations above, it is evident that when sensors are placed around the payload, attention should be paid to the appearance of high-speed airflow above the booster. In addition, consideration should be given to sudden pressure changes at the payload’s head, caused by the interference of shock waves and expansion waves.

**Figure 10:**
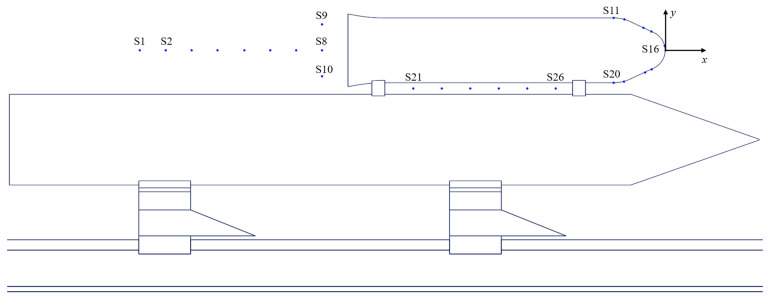
Monitoring points’ distribution.

To capture the flow field characteristics of the rocket sled during variable acceleration sliding and guide the sensor layout of the test system, it is necessary to monitor the changes in pressure around the payload over time. Multiple monitoring points are set up at the head, tail, and between two connectors to obtain the pressure distribution. The distribution of monitoring points is shown in Figure 10. The tip of the payload’s head serves as the origin, and these points’ relative coordinates are listed in the Table 1. $X=x_{t}/x_{0}$ ($x_{t}$ is the streamwise distance measured from the coordinate axis, $x_{0}$ is the length of the payload), $Y=y_{t}/y_{0}$ ($y_{t}$ is vertical distance measured from the coordinate axis, $y_{0}$ is the diameter of the payload). S1~S10 monitor the pressure change at the trail of the payload. S11~S20 monitor the pressure change on the surface of the payload’s head, with S16 positioned at the tip. S21~S26 observe the pressure change between the payload and the booster. The pressure $P^{*}$ is non-dimensional, i.e., $P^{*}=P_{t}/P_{0}$ (where $P_{t}$ is the actual pressure monitored at each point, $P_{0}$ is the standard atmospheric pressure, 101,325 Pa).

**Figure 11:**
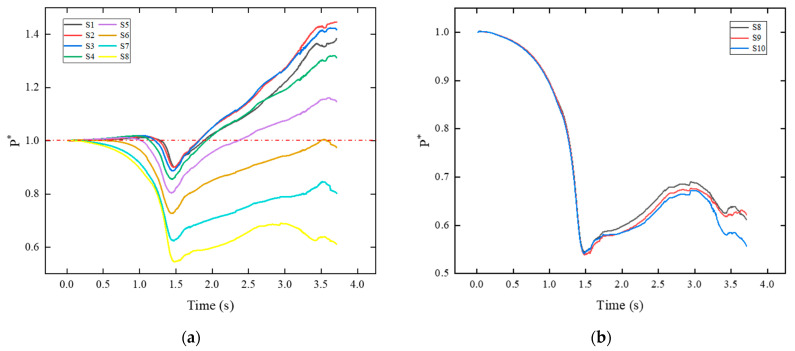
Pressure variation at monitoring points: (**a**) S1–S8; (**b**) S8–S10.

Two graphs in Figure 11 show the pressure variation of the monitoring points situated at the trail of the payload. In Figure 11a, it can be observed that during the initial phase of sliding, the pressure at point S8 (closest to the tail of the payload) decreases first. Subsequently, the pressure at other points decreases, while the influence range of the payload wake vortex gradually expands. The minimum pressure of all monitoring points occurs before 1.5 s. As the sliding progresses, the value $P^{*}$ at S1~S5 exceeds 1, indicating that these areas are almost unaffected by the wake vortex. Figure 11b shows the pressure at these three points largely overlaps from 0 s to 1.5 s, and the peak intensity of the wake vortex appears at 1.5 s. As time passes, differences in pressure emerge at these points. The pressure distribution along the vertical direction near the tail becomes uneven. Additionally, the pressure at points S8, S9, S10 decreases after 3.0 s, a phenomenon also observed at the monitoring points in Figure 11a. This is due to the decreasing extent of the low-speed region behind the payload, leading to an increase in core intensity.

**Figure 12:**
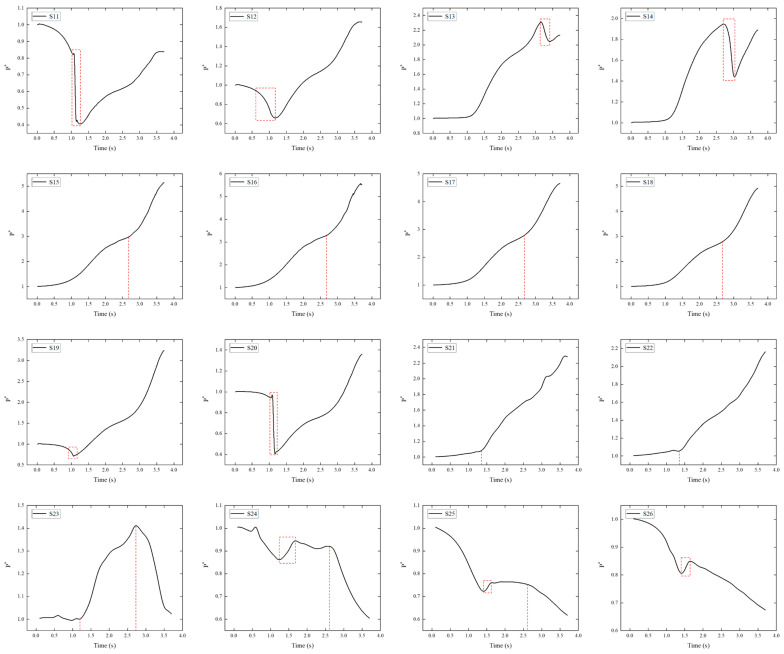
Pressure variation at S11~S26.

Figure 12 shows the pressure of monitoring points S11~S26. At around 1.1 s, the pressure suddenly decreases at S11, S12, S19, and S20 (highlighted by red dashed boxes). This is because of the change in the shape of the payload, which causes expansion waves to start to appear at the payload’s head (as can be seen in Figure 9), leading to a pressure decrease. Points S11 and S20 are closer to the smooth section, where the pressure drop is more pronounced. At the later stages of sliding, due to the impact of shock waves and stagnant air flow at the connector, the values $P^{*}$ at point S20 gradually increase. At points S13 and S14, there is a rapid increase in pressure around 1.2 s. At this time, a shock wave appears at the payload’s head, followed by a sudden decrease in pressure. There is a noticeable change in the curvature of the model, so it is speculated that an expansion wave will form around at 3.0 s and interfere with the head shock wave. At around 2.7 s, a significant increase in pressure occurs at points S15, S16, S17, and S18 (marked with red dashed lines). It can be observed that at this time, the shock waves generated by the booster’s head are impacting the payload’s head. Furthermore, observations from points S11, S12, and S13 reveal a surge in pressure after 2.7 s. This indicates an interference between the shock waves generated by the booster’s head and the payload’s head, affecting the pressure distribution across the entire payload’s head.

The pressure distribution at monitoring points located in the intermediate section between the payload and the booster are shown in the last six images in Figure 12. The pressure continues to increase at points S21 and S22, attributed to the obstruction effect of the rear connector on the airflow, leading to a gradual rise in pressure. It is worth noting that the increasing trend is not linear. At point S23, the pressure does not continuously increase; instead, it gradually decreases around 2.6 s. At this moment, the pressure at points S24 and S25, situated within the low-pressure vortex region, begins to decline, indicating an expansion and intensification of the low-pressure vortex area. Around 1.5 s, the pressure at points S24, S25, and S26 starts to rise (highlighted by red dashed boxes), implying a temporary decrease in low-pressure vortex intensity. At this point, the rocket sled reaches supersonic speeds, and shock waves from the front slipper impact the area between the two connectors, leading to an increase in pressure (the pressure at points S21 and S22 also suddenly rises). As the speed increases, the shock wave angle decreases, reducing the impact on the monitoring points closer to the front slipper.

**Figure 13:**
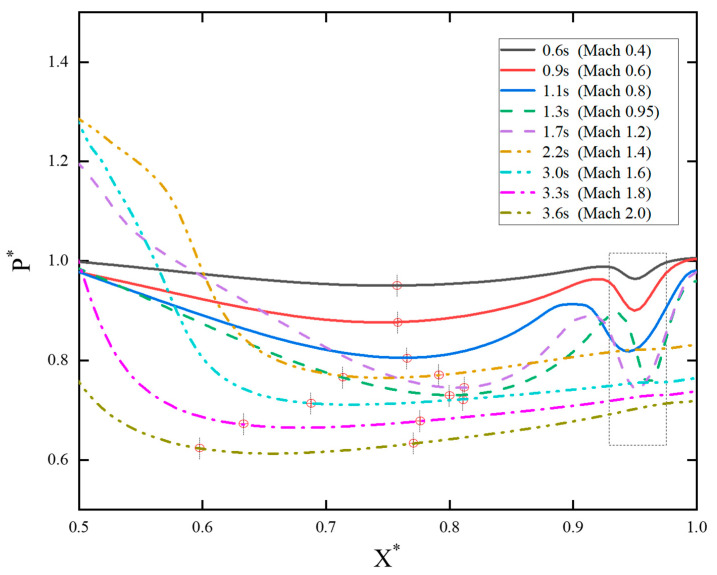
Pressure distributions on the upper surface of the booster.

Figure 13 illustrates the pressure distribution on the upper surface of the booster, between two connectors. $X^{*}=X_{l}/X_{0}$ ($X_{l}$ represents the distance of the monitoring point from the rear connector, while $X_{0}$ represents the distance between two connectors. $X^{*}=1$ denotes the position at the front connector). The positions where the lowest pressure occurs at different sliding times are marked with vertical dashed lines and red circles. As the sliding speed increases, the pressure generally decreases at various points. However, in the transonic phase ($X^{*}=0.5\sim 0.6$), the pressure significantly increases, surpassing the levels observed during the subsonic phase. When the speed stabilizes in the supersonic phase, the pressure gradually decreases. During the initial sliding period from 0.6 s to 1.1 s (Ma = 0.4~0.8), the position of the low-pressure vortex remains nearly unchanged. However, from 1.1 s to 1.7 s (Ma = 0.8~1.2), the low-pressure vortex moves forward. During the later stages of sliding from 2.2 s to 3.6 s (Ma = 1.4~2.0), the position of the lowest pressure becomes less distinct. Instead, the interval ($1P_{\min}^{*}\sim 1.05P_{\min}^{*}$) is chosen to represent the low-pressure region. The low-pressure vortex shifts rearward with increasing sliding velocity, and its range expands continuously. The area outlined by the black dashed box in Figure 13 represents the low-pressure region, obstructed by the front connector. However, from 2.2 s to 3.6 s, this feature is no longer prominent. The low-pressure vortex region behind expands to the vicinity of the front connector.

Based on the simulations above, it can be found that during the transonic phases, the position of the inter-connector vortex may shift forward slightly. However, as the rocket sled reaches supersonic velocity, the influence range of the vortex expands continuously. The variation in vortex position, caused by changes in sliding velocity, needs special consideration when capturing the connector vortex.

### 4.2. Aeroacoustic Characteristics of Variable Acceleration Rocket Sled

**Figure 14:**
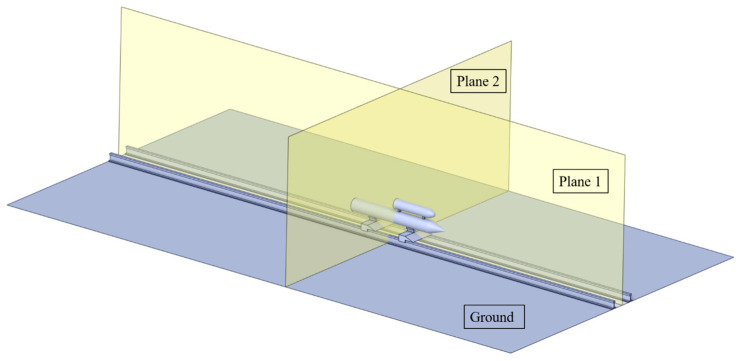
Planes set up for aeroacoustic analysis.

To capture the distribution of aeroacoustic characteristics around the rocket sled, several receivers are placed on two planes, as shown in Figure 14. Plane 1 is the symmetry plane, monitoring the aeroacoustic distribution in the direction parallel to the flow. Plane 2 is perpendicular to the velocity direction and intersects with the middle of the rocket sled, monitoring the aeroacoustic distribution in the vertical flow direction.

**Figure 15:**
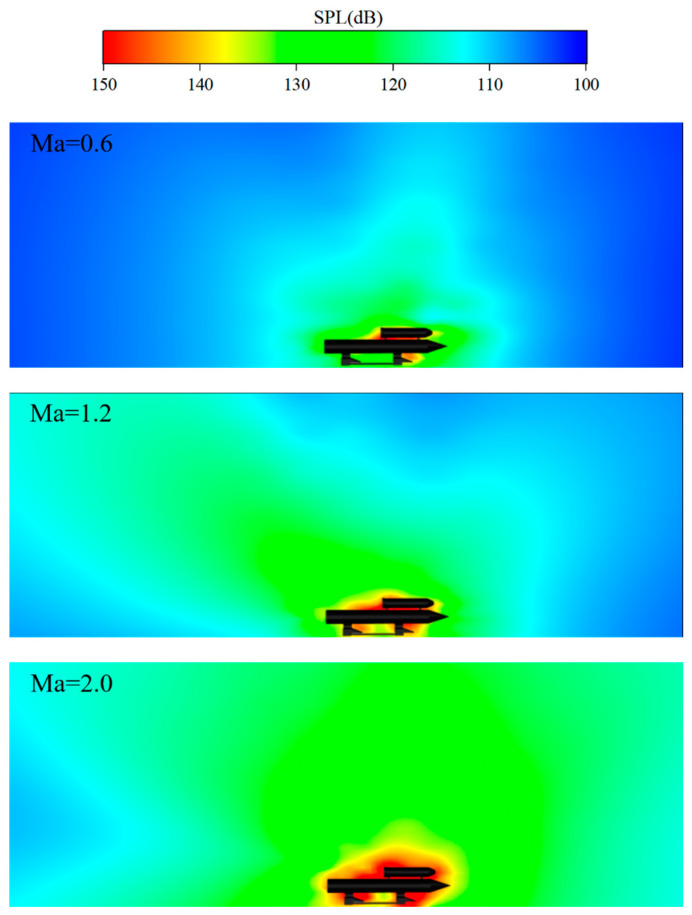
Sound pressure level on the symmetry plane.

Firstly, four thousand aeroacoustic receivers (100 horizontal, 40 vertical) are uniformly placed on Plane 1 to obtain an instantaneous sound pressure level (SPL) contour at 0.9 s (Ma = 0.6), 1.7 s (Ma = 0.6), 3.6 s (Ma = 2.0), as shown in Figure 15. At the sliding speed of Mach 0.6, a high SPL appears in the middle section between the payload and the booster, as well as in the front slipper area. Moreover, the noise propagates upwards and backwards. According to Ref. [15], the vortices between the two connectors of a subsonic rocket sled indicate potential vortex-induced aeroacoustics. This finding aligns with the regions of the high SPL distribution observed in this study. At a Mach number of 1.2, the high SPL area expands (particularly at the rear of the payload), with a noticeable trend of noise propagating backward. When the velocity reaches Mach 2.0, the high SPL area nearly covers the entire rocket sled. It is important to reinforce structural protection and reasonably place sensors to prevent the impact causing by high SPL noise. Additionally, in the supersonic phase, there is a low SPL region between the two slippers.

**Figure 16:**
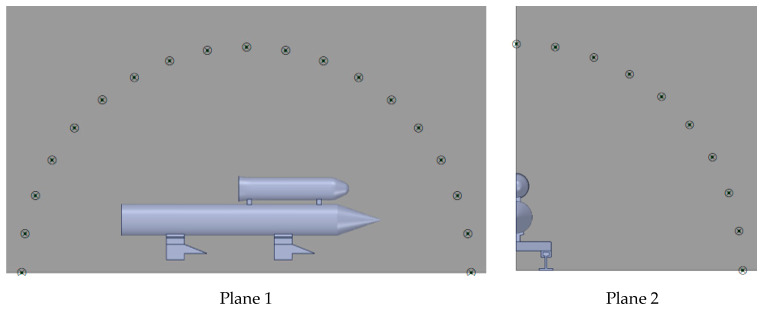
Receivers set for aeroacoustic analysis (receivers are marked with × and circles).

**Figure 17:**
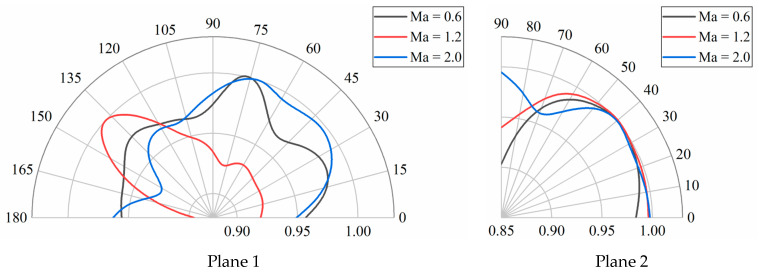
Polar diagrams in two planes.

To obtain the aeroacoustic directivity near the rocket sled, several receivers are set on two planes. As Figure 16 shows, nineteen receivers are evenly distributed along a semicircle with a diameter four times the length of the payload on Plane 1. Due to the symmetry of the rocket sled, ten receivers are placed on Plane 2, distributed along a quarter circle with the same diameter as that in Plane 1. The sound pressure from these receivers is processed, and the aeroacoustic directivity in two directions is depicted in Figure 17. The radius *r* in these diagrams is the dimensionless sound pressure, $r=P_{r}/P_{r,\max}$ (where $P_{r}$ refers to the sound pressure recorded at each receiver, and $P_{r,\max}$ is the maximum sound pressure observed at the same sliding velocity). In Plane 1, an angle coordinate of 180° represents the opposite direction to the sliding direction, while 90° represents the upward vertical direction in Plane 2.

In Plane 1, when the rocket sled accelerates to Mach 0.6, the noise propagates along three different directions, with the main direction pointing at 80° and two secondary directions pointing at 30°~45° in the front and 125°~140° in the rear. When the velocity reaches Mach 1.2, the noise directivity becomes more pronounced, pointing towards 140°. At a Mach number of 2.0, the noise mainly propagates towards the direction of 30°~75°. In the direction perpendicular to the velocity, the noise directivity is almost the same, primarily pointing towards 20°~50°. In addition, the trend of noise propagation along the 90° direction becomes significant with the increase in sliding velocity. When the Mach number is 2.0, due to the high SPL region around the rocket sled, the noise directivity is not pronounced. Sensors susceptible to interference need to avoid directions with strong noise directivity. For example, at Mach 0.6, avoid placing sensors in the direction of 75°; at Mach 1.2, avoid the direction of 135°; and at Mach 2.0, avoid the direction between 30° and 75°.

## 5. Conclusions

The aerodynamic and aeroacoustic characteristics of the variable acceleration rocket sled have been analyzed. It can be concluded that in the initial stage of sliding, the range of the payload wake vortex gradually expands, peaking at 1.5 s, and then decreases over time. In the subsonic stage, the position of the vortex between the payload and the booster remains stable. Shock waves and expansion waves begin to form in the head–slippers–ground region, leading to complex interference. In the transonic stage, shock waves from the front slipper cause the vortex to move forward. In the later stage of sliding, the vortex gradually moves backward, and the influence range continues to expand. When sensors are placed at the tail of the payload, attention should be paid to the sudden emergence of a high-speed airflow over the booster. In addition, the variation in vortex position caused by the change in sliding velocity must be considered.

In the later sliding stage, the rocket sled is enveloped by regions of high sound pressure levels. In the subsonic and transonic stages, the noise directivity of the rocket sled is more pronounced. The noise mainly propagates towards 80° and 140°, respectively. However, in the supersonic stage, particularly in the later sliding stage, the directivity is no longer prominent. Sensors should be placed reasonably to minimize interference from aerodynamic noise.

All these conclusions can provide valuable insights into the sensor layout for variable acceleration rocket sled tests. Future research will be conducted on a winged payload model and the separation of the payload from the booster.

## Figures and Tables

**Table 1:** Coordinates of the monitoring points.

Point No.	X	Y	Point No.	X	Y
S1	−1.624	0	S14	−0.043	0.261
S2	−1.544	0	S15	−0.002	0.064
S3	−1.464	0	S16	0	0
S4	−1.384	0	S17	−0.043	−0.261
S5	−1.304	0	S18	−0.062	−0.304
S6	−1.224	0	S19	−0.128	−0.429
S7	−1.144	0	S20	−0.160	−0.446
S8	−1.064	0	S21	−0.778	−0.529
S9	−1.064	0.357	S22	−0.690	−0.529
S10	−1.064	−0.357	S23	−0.602	−0.529
S11	−0.160	0.446	S24	−0.514	−0.529
S12	−0.128	0.429	S25	−0.426	−0.529
S13	−0.0681	0.311	S26	−0.338	−0.529

## Data Availability

The data presented in this study are available on request from the corresponding author.
